# A relational database to identify differentially expressed genes in the endometrium and endometriosis lesions

**DOI:** 10.1038/s41597-020-00623-x

**Published:** 2020-08-28

**Authors:** Michael Gabriel, Vidal Fey, Taija Heinosalo, Prem Adhikari, Kalle Rytkönen, Tuomo Komulainen, Kaisa Huhtinen, Teemu Daniel Laajala, Harri Siitari, Arho Virkki, Pia Suvitie, Harry Kujari, Tero Aittokallio, Antti Perheentupa, Matti Poutanen

**Affiliations:** 1 Institute of Biomedicine, Research Center for Integrative Physiology and Pharmacology, University of Turku, 20520 Turku, Finland; 2 Department of Obstetrics and Gynecology, University of Turku, and Turku University Hospital, 20014 Turku, Finland; 3 Institute of Biomedicine, Research Center for Cancer, Infections and Immunity, University of Turku, 20520 Turku, Finland; 4 Department of Pathology, Turku University Hospital, 20521 Turku, Finland; 5 Department of Mathematics and Statistics, University of Turku, 20014 Turku, Finland; 6 Institute for Molecular Medicine Finland (FIMM), University of Helsinki, 00014 Helsinki, Finland; 7 Department of Neurology, Faculty of Medicine, University of Turku, 20014 Turku, Finland; 8 Turku Bioscience Centre, University of Turku and Åbo Akademi, Turku, Finland; 9 Institute of Medicine, Sahlgrenska Academy, 405 30 Gothenburg University, Gothenburg, Sweden

**Keywords:** Transcriptomics, Endocrine reproductive disorders, Translational research

## Abstract

Endometriosis is a common inflammatory estrogen-dependent gynecological disorder, associated with pelvic pain and reduced fertility in women. Several aspects of this disorder and its cellular and molecular etiology remain unresolved. We have analyzed the global gene expression patterns in the endometrium, peritoneum and in endometriosis lesions of endometriosis patients and in the endometrium and peritoneum of healthy women. In this report, we present the EndometDB, an interactive web-based user interface for browsing the gene expression database of collected samples without the need for computational skills. The EndometDB incorporates the expression data from 115 patients and 53 controls, with over 24000 genes and clinical features, such as their age, disease stages, hormonal medication, menstrual cycle phase, and the different endometriosis lesion types. Using the web-tool, the end-user can easily generate various plot outputs and projections, including boxplots, and heatmaps and the generated outputs can be downloaded in pdf-format.

Availability and implementationThe web-based user interface is implemented using HTML5, JavaScript, CSS, Plotly and R. It is freely available from https://endometdb.utu.fi/.

## Abstract (summary)

**Table:** 

Measurement(s)	RNA • differential expression analysis data • endometriosis
Technology Type(s)	Microarray Analysis • digital curation
Factor Type(s)	age • disease stage • menstrual cycle phase • hormonal medication • endometriosis lesion type • endometrium
Sample Characteristic - Organism	Homo sapiens

Machine-accessible metadata file describing the reported data: 10.6084/m9.figshare.12800642

## Background & Summary

Endometriosis is a common, chronic, and benign estrogen-dependent gynecological disorder associated with inflammation, pelvic pain, and reduced fertility in affected women. The prevalence of endometriosis in reproductive aged women varies between 5–10%, while the frequency in women with pelvic pain with or without infertility is between 50–60%^1–3^. Endometriosis is characterized by the presence of endometrium-like tissue growing in ectopic locations outside the uterine cavity. The ectopic lesions respond to ovarian derived steroid hormones, with a tendency for recurrence after surgical treatment^1^. The etiology and pathogenesis of endometriosis is multifactorial and still poorly understood, and the current treatment strategies, including pharmacological therapies, are not curative and often do not alleviate the pain symptoms^4,5^.

In classifying endometriosis, the proposed disease classification by the American Society of Reproductive Medicine (ASRM) is the most widely used. It provides a standard form for reporting pathological findings, together with a numeric value for the disease status^6^. The ASRM classification assigns points based on the spread of the endometriosis tissue, its infiltration depth in ectopic sites, and the areas of the body affected.

In this report, we present the EndometDB, an interactive web-based user interface easily applicable for browsing the gene expression database of collected samples without the need for computational skills. The patient features associated with the lesions within the EndometDB can be used as stratifying factors when investigating the gene expression patterns. Endometriosis type can be defined also by its clinical appearance and by which area of the pelvis or abdomen the lesions affect: Ovarian endometrioma, peritoneal endometriosis lesion, and deep infiltrating lesion, and all these features are available to be linked to the mRNA expression data in the EndometDB. Similar to the eutopic endometrium, endometriosis progression is highly dependent on sex steroid action, and the lesion growth is highly dependent on estrogen stimulus^7^. Due to the strong sex steroid dependency, hormonal treatments, e.g. with oral contraceptives, that suppress ovarian steroid hormone action are used to reduce the lesion growth and manage the pain symptoms. In the EndometDB, the gene expression can be associated with the menstrual cycle, hormonal medication status of the affected women and the ASRM disease classification.

Comparing the gene expression profiles of disease tissue to that of a normal healthy tissue is a powerful approach to understand the underlying cellular events in the etiology of any disease^8^. Accordingly, gene expression changes associated with endometriosis have also been analyzed in previous studies using various microarray platforms by comparing the endometriosis lesions with eutopic endometrial tissues^9–13^, or by comparing the endometrium of the patients to that of healthy controls^14–16^. All these studies have offered some essential understanding into the transcriptional differences related to endometriosis, however, only a limited number of samples were included due to various constraints, with samples size ranging from between 6 and 25. To address this limitation, the Endomet database includes the most extensive collection of lesions so far analyzed for genome-wide mRNA expression. Furthermore, several studies have analyzed only the ovarian lesions^10,17^, largely due to the ample availability of such samples.

**Fig. 1:**
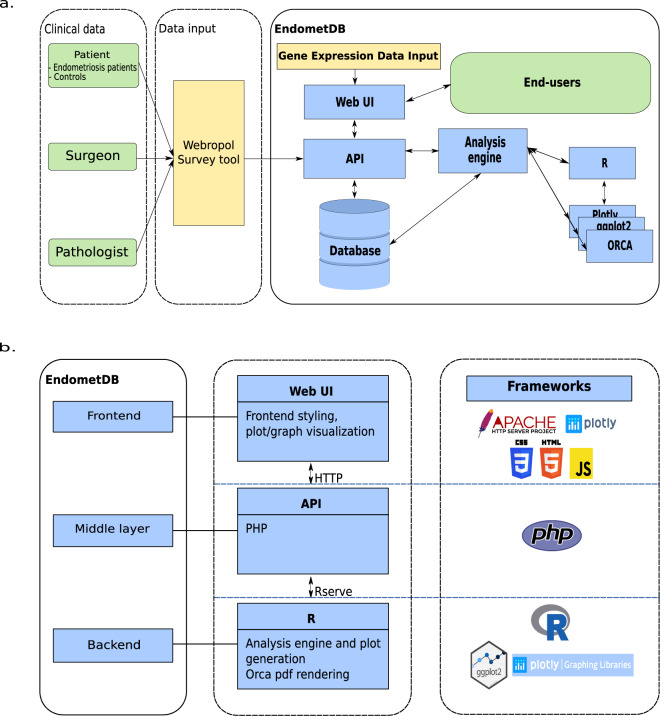
**a**) Schematic overview of the EndometDB used for transcriptomic analysis. The questionnaire data is collected through the Webropol survey system and imported into EndometDB via an automated service layer API. The numerical results of the biomedical samples analyzed are uploaded into the system together with the sample information. The uploading is done through the web user interface. Analytical functions are available through the analysis engine and can be used to query the whole data set. (**b**) EndometDB web graphical user interface (GUI) analysis process. The GUI, through a client, communicates with the analysis engine through an API layer implemented with PHP. The analysis engine analyzes the data using the R programming language and Plotly graphing library with the ggplot2 R package is used to generate the plots that are transferred back to the GUI.

Overall, the field of endometriosis study is primed to further characterize and describe specific pathways involved in the disease and there is still a need for more systematic and comprehensive analysis of the gene expression patterns across different types of endometrial lesions as the different forms of endometriosis may express different markers/genes differently^18^. Analyzing different lesion types could aid in the identification of the potential diversity in the etiology of the different lesion types. As an example, using the data included in the EndometDB we identified Secreted frizzled-related protein 2 (SFRP2) to be a gene with high expression in endometriosis compared to the endometrium. The protein was shown to be a novel lesion border marker in histological sections, and as a secretory protein it has a potential to serve also as a serum biomarker^19^. The current version of the EndometDB consists of structured mRNA expression information from 115 patients and 53 controls (Table 1), with the data available from 190 lesions of different types. The EndometDB can be explored through several patient factors, such as age, cycle phase, disease stage and hormonal medication status. The tissues are histologically confirmed, and the mRNA expression on patient and healthy endometrium and peritoneum can also be analyzed. The database integrates clinical data (Fig. 1a) and tissue types (endometrium, peritoneum and the different endometriosis lesion types) with the transcriptomic data (>48000 measured), and the graphical user interface (GUI) allows easy access to the curated data. The entire transcriptomic data in the EndometDB can be explored all at once or in subsets. The users can choose whether they perform the expression analysis based on all expression data of over 24000 genes or on only the genes of interests (Fig. 1b). The EndometDB with detailed transcription profiles of eutopic and ectopic endometrium is a valuable tool for identifying potential biomarkers and treatment targets, and to gain novel information on the gene expression networks associated with the lesion growth. This in turn could aid the development of novel diagnostic and prognostic markers predictive of endometriosis and to understand the pathogenesis of endometriosis better.

**Table 1:** Clincal characteristics of patients with endometriosis and healthy controls used in the present study.

Parameter	Patient group (n = 115)	Control group (n = 53)
Mean age (SD, range)	32 (6.8, 20–48)	39 (4.7, 24–48) *** ^a^
Median BMI^b^ (range)	23 (17.3–40.6)	24 (18.9–41.2)
**rAFS stage**		
I	15 (8.9%)	NA
II	15 (8.9%)	NA
III	26 (15.5%)	NA
IV	56 (32.2%)	NA
Missing Data	3 (1.8%)	NA
**Indication for surgery**		
Pain	71 (42.3%)	NA
Infertility	6 (3.6%)	NA
Both pain and infertility	22 (13.1%)	NA
Clinical finding in gynecological examination	15 (8.93%)	NA
Not recorded	1 (0.6%)	NA
**Menstrual cycle phase**		
Proliferative	19 (11.3%)	14 (8.3%)
Secretory	26 (15.5%)	12 (7.1%)
Menstrual	6 (3.6%)	1 (0.6%)
Inactive, atrophic or insufficient	51 (30.4%)	18 (10.7%)
Missing Data	13 (7.7%)	8 (4.8%)

Note: BMI = Body mass index; NA = not applicable; NS = not significant, ^a^*** < 0.0001, Two-sample t-test, ^b^BMI missing 2 (2%) in the patient group and 2 (4%) in the control group.

## Methods

### Ethics approval and informed consent

The study protocol was approved by the Joint Ethics Committee of Turku University and Turku University Central Hospital in Finland and registered in Clinical Trials.gov as trial number NCT01301885. Prior to surgery a written informed consent for participation in the study was required from all the study subjects. All specimen collected are part of the Auria biobank sample collection (https://www.auria.fi/biopankki/en/index.php?lang = en). The sample collection protocol closely resembles those recommended by World Endometriosis Research Foundation Endometriosis Phenome and Biobanking Harmonization Project and the Endometriosis Phenome and Biobanking Harmonization Project WERF/EpHECT^20–24^, despite carrying out the collection before those recommendations were published.

### Study design

This study was conducted at the Department of Obstetrics and Gynecology Turku University Hospital, University of Turku, Finland, and the Institute of Biomedicine, Research Centre for Integrative Physiology and Pharmacology, University of Turku, Finland. Samples of endometriosis, eutopic endometrium and peritoneum were collected from endometriosis patients, at 4 different hospitals in Finland and healthy tissues from the endometrium and peritoneum were obtained from women undergoing laparoscopic tubal ligation at the Turku University Hospital, University of Turku, Finland. A definitive diagnosis was reached through laparoscopy or laparotomy, and endometriosis was further confirmed by histopathological evaluation of obtained biopsies. Endometriosis was excluded by laparoscopy during tubal sterilization in healthy women. The menstrual cycle stage was determined at the day of sampling using a questionnaire, endometrial histology, and serum progesterone concentration. Three different endometriosis sample subtypes were collected for transcriptional analysis: 1) deep infiltrating endometriosis lesions (DiE), including deep rectovaginal (REV), sacrouterine ligament lesion (SuL), intestinal endometriotic lesions (DiEIn) and deep endometriotic lesions in the bladder (DiEB); 2) peritoneal endometriosis lesions, including red peritoneal endometriotic lesion (PeLR), black peritoneal endometriotic lesion (PeLB) and white peritoneal endometriotic lesion (PeLW); and 3) ovarian endometrioma samples (OMA). Endometrium samples from both patients (PE) and healthy controls (CE) were collected, as well as peritoneum samples from both healthy controls (CP) and patients (PP). Patient characteristics are presented in Table 1, and the samples used in the transcriptomic analysis are described in Table 2. All tissues used for mRNA analyses were snap-frozen and stored in liquid nitrogen within 10 min, until used.

**Table 2:** Current samples in gene expression profiling.

Samples					
Tissue type	Total	Proliferative phase	Secretory phase	Hormonal medication	Others
Control endometrium	43	14	12	10	7
Patient endometrium	101	16	28	43	14
Ovarian endometriosis	28	7	9	7	5
Peritoneal endometriosis	76	13	15	37	11
Deep endometriosis	86	9	16	48	13
Control peritoneum	24	3	6	12	3
Patient peritoneum	38	4	9	15	10
**rAFS stage**					
I–II	30	3	8	14	5
III	26	7	5	10	4
IV	56	9	12	26	9
Missing	3		1	1	1

### PostgreSQL relational database

To implement the EndometDB, we used PostgreSQL (https://www.postgresql.org/), an open-source object-relational database management system (ORDBMS) that allows for the handling of workloads ranging from small-machine application to large internet scale applications with many concurrent users. The PostgreSQL database stores information and metadata on a Linux server that efficiently and securely deals with computational demands. We implemented an application programming interface (API) with the EndometDB to allow for smooth communication between server and clients (web browser on computers, tablets, etc.) which is specifically adapted for sending SQL queries to the database and serving the results in standardized format to the client. In addition, it interfaces with the analysis engine which itself sends custom queries to the database to retrieve measurement data for statistical analysis and visualization. This stable architecture can be also extended to future needs arising from new functionalities developed on different platforms.

### Web-based graphical user interface

**Fig. 2:**
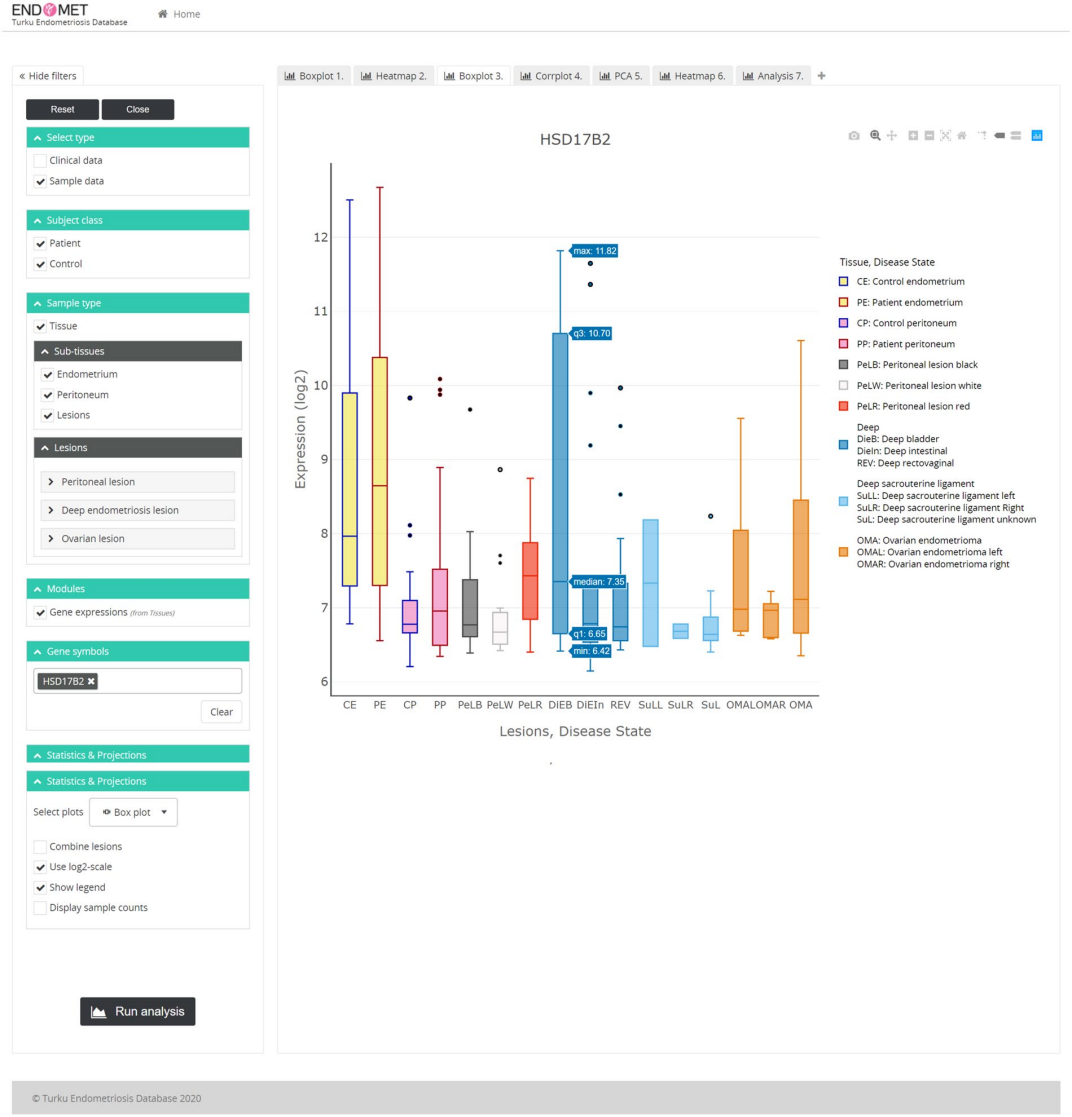
EndometDB GUI. Screenshot of the EndometDB user interface showing tabbed browsing functions (on the left) and an example boxplot (HSD17B2) as an output of a gene analyzed (on right). The browsing functions include controls for interacting with the filters such as the clinical data (age, menstrual cycle phase, hormonal medication, disease stage), sample data (tissue type; endometrium, peritoneum, endometriosis lesions) and plots and projections. The different color boxplot represents the different tissues and lesions for both the healthy women and patients. The colors make it easier to distinguish between the different tissues and lesions as well as between healthy controls and patients.

The EndometDB is implemented on an Ubuntu Linux system and incorporates a GUI that utilizes HTML5, JavaScript, PHP, and R as the main programming languages. The GUI also uses jQuery, Plotly.js, and CSS for the frontend styling, and the graph visualizations are generated with the Plotly R open source graphing library. The GUI was developed to accommodate both physicians and researchers with the two modes separated by user-based authentication. The publicly available part features an informational site with pages for research overview, team members, collaborating partners and contact details, as well as a comprehensive set of analytical tools for data visualization and basic statistical assessment of transcriptomic data. The GUI allows users, through a client, to send requests to the analysis and visualization engine via an API layer implemented with PHP. The analysis engine is implemented as a S3 R package and utilizes several R packages for statistics and graphical output, in particular ggplot2^25^, Plotly and HTML widgets, to generate a JSON representation of the plots which is then transferred via the Plotly JavaScript Open Source Graphing Library back to the GUI where it is displayed in the user’s browser. We also used the Open source Report Creator App R Package (ORCA) on the backend to allow the user to render the generated plot to PDF (Fig. 1b). List of programming language and URL in Table 3. The design of the EndometDB web graphical user interface enables users to search and analyze the data in the database without the need for computational skills, and can search data related to a gene(s) of interest by typing or copy pasting the gene symbol(s) into the designated area (Fig. 2).

**Table 3:** List of programming languages.

	Programming languages		URL
Main programming language	HTML 5		https://www.w3.org/html/, https://whatwg.org/
	JavaScript		https://developer.mozilla.org/en-US/docs/Web/JavaScript
	PHP		https://php.net/
	R		https://www.r-project.org/
Frontend styling	jQuery		https://jquery.com/
	Plotly.js		https://plot.ly/javascript/
	CSS		https://developer.mozilla.org/en-US/docs/Web/CSS
Graph visualization	Plotly R graphing library		https://plot.ly/r/
API	PHP		https://php.net/
Backend	R		https://www.r-project.org/
	Analysis engine	Plotly graphing library	https://plot.ly/r/
		ggplot2 R package	https://www.rdocumentation.org/packages/ggplot2/versions/3.1.1
		ORCA	https://www.rdocumentation.org/packages/plotly/versions/4.9.0/topics/orca

**Fig. 3:**
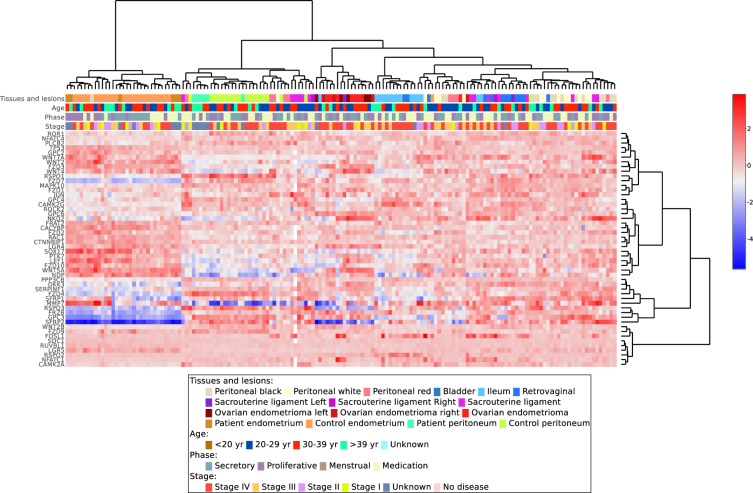
Example output of unsupervised hierarchical clustering analysis generated via the EndometDB GUI. Example of unsupervised hierarchical clustering analysis of mRNA expression of the differentially expressed WNT pathway genes (Online-only Table 1) in all the sample groups. The different clinical features of the samples (lesion/tissue type, age of the patients with pre-selected grouping, hormonal stage, and disease stage) are attached to the heatmap. Canberra distance metric with Ward’s clustering method was applied showing clusters corresponding to lesions and tissue types. The dendrogram on the x-axis shows the hierarchical relationship between the tissues and lesion as well as the cycle phase and disease stage. While the dendrogram on the y-axis shows the measure of similarities in the activation levels of the WNT signaling pathway genes. The colors represent different tissues and lesions from both healthy controls and patients.

The GUI incorporates different techniques and analytical methods to analyze transcriptomic data. The EndometDB GUI allows users to browse and view data in tabbed sections, rather than having to open multiple pages that take up computing resources (Fig. 2). One of the many techniques and analysis methods the EndometDB GUI provides, relies on filter-based data mining which allows mRNA expression of genes of interest in various endometriosis lesion types, and in the endometrium and peritoneum from both controls and patients, and clinical features such as age, menstrual cycle phase, hormonal medication, and disease stage which can be used for stratification, be displayed for instance, with boxplots (Fig. 2) which shows the range of the data distribution. Users can also choose to simultaneously compare expression patterns between different genes of interests or pathway genes. These comparisons can then be displayed e.g. with a heatmap (Fig. 3) and be summarized by either the median or mean, and the user may further center the data using the gene or lesion. Users can display the heatmaps using different unsupervised hierarchical clustering algorithms (Complete linkage, Single linkage, Average linkage, or Ward’s method), and with predefined distance methods (Euclidean, Canberra, Manhattan, Maximum, or Minkowski).

**Fig. 4:**
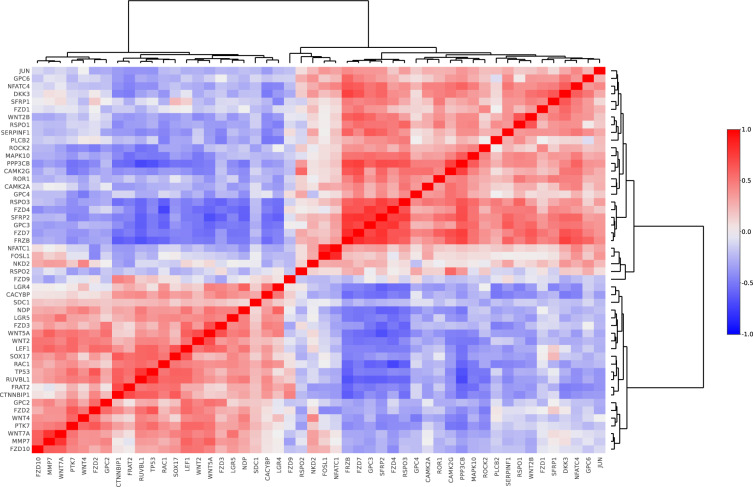
Example of correlation heatmap from the EndometDB generated via the GUI. Correlation heatmap after hierarchical clustering of WNT pathway genes (Online-only Table 1) with Pearson correlation method and Ward’s clustering method. Clustering dendrogram on both axes show the measure of the relationship between the genes.

Clinical features such as age, menstrual cycle phase, hormonal medication, and disease stage can also be used as contrast in the hierarchical clustering to show how groups of genes relate to these clinical features. Users can also use the correlation heatmap feature (Fig. 4) with the most used correlation methods (Pearson, Spearman, and Kendall), to show the correlation matrix between two discrete dimensions. The correlation heatmap can also be clustered using the most used hierarchical clustering methods to analyze how genes of interest correlate with each other in the different lesions or tissues. These methods provide information about the involvement of analyzed genes in the connected biological processes.

**Fig. 5:**
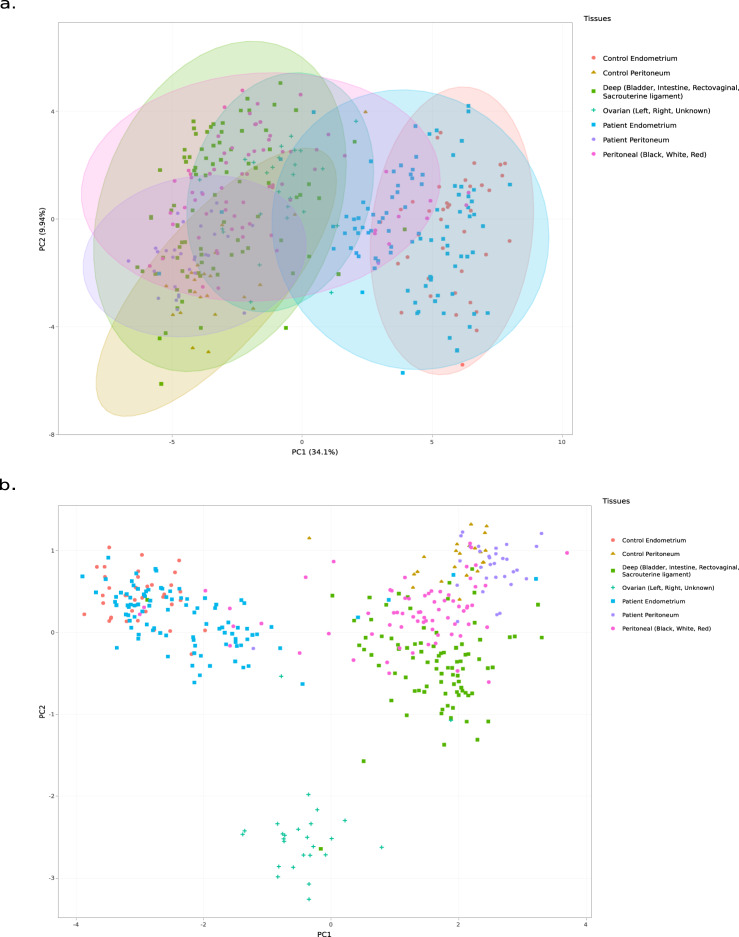
Example of projection outputs from the EndometDB generated via the GUI. (**a**) PCA analysis of mRNA expression of the differentially expressed WNT pathway genes in all the sample groups. Samples are colored by tissue types, and the confidence ellipses with 95% confidence level for the expression in various tissue types are generated using the EndometDB GUI. The PCA separates control and patient endometrium from the three subtypes of endometriosis lesions. (**b**) LFDA analysis of WNT pathway genes with raw eigenvectors metric colored by tissues. In addition to separating endometriosis lesions from endometrium, LFDA separates ovarian endometriosis from peritoneal and deep endometriosis. The list of WNT pathway genes used in these analyses are listed in Online-only Table 1.

To further investigate similarities in the gene expression, e.g., between the various sample types or to identify gene clusters to generate further hypotheses, the EndometDB web GUI tool includes three dimensionality reduction methods: Principal Component Analysis (PCA, Fig. 5a), Local Fisher Discriminant Analysis (LFDA, Fig. 5b) and Multidimensional scaling^26^. Users can also choose to display the scree plot that shows how much each of the PCA components accounts for the total variance in the gene expression data^26^. The principal components in the scree plot are listed by decreasing order of contribution to the total variance and the bars in the output show the proportion of variance represented by each component. Users are can also choose to color the PCA using predefined groups such as tissues, subject class, disease stage, menstrual cycle phase, and age as well as display the confidence ellipses that shows the variability in the data (Fig. 5a). The confidence ellipse label can be viewed when users mouse-over the generated plot or by selecting the label ellipses checkbox.

In the EndometDB the LFDA may be used to find a linear combination of genes that characterize or separate two or more sample classes, and simultaneously maintain the local structure of the expression data. Three metric types can be used in the EndometDB with LFDA (Raw eigenvectors, Weighted eigenvectors, and Orthonormalized), and colored by predefined groups (Fig. 5b). MDS is a technique used in detecting and visualizing meaningful underlying dimensions that allows for researchers to explain observed similarities or dissimilarities (distances) between the investigated samples^26^. Defined distance methods such as Euclidean, Canberra, Manhattan, Maximum, and Minkowski can be used with MDS for visualization of the similarities or dissimilarities and colored by these predefined groups (tissues, subject class, disease stage, menstrual cycle phase, and age).

### Interactive visualization

The interactive visualization is implemented using the Plotly open source JavaScript graphing library (https://plot.ly/javascript/). This provides the EndometDB GUI users with interactive features (Fig. 2). The interactive visualization has four components: 1) Hover data, which allows users by mouse-over to view values within the graph; 2) Click data, which allows users to click on points in the graph; 3) Selection data, which allows users to choose the lasso or rectangle tool in the graph menu bar, and then select points of interest in the graph and 4) Zoom and relay out data, which allows the users to click and drag the graph to zoom or click the zoom buttons in the graph’s menu bar. These components enable the user, by moving the mouse pointer over tiles of the heatmap, or over row/column labels, or the box plot, to display additional information. The EndometDB GUI sidebar (Fig. 2) contains controls for interacting with the visualization that allow users to select filters such as the clinical data (age, cycle phase, hormonal medication, disease stage), sample data (tissue type; endometrium, peritoneum, endometriosis lesions) and various types of plot outputs and statistics. The user can further interact with the generated outputs by clicking on the legend (on the right side of the figure) to relay out the data within the plotted graph.

### Generating the transcriptomics data by microarrays

The global transcriptomics data of all the tissue specimens presented in the EndometDB were generated on the Sentrix® Illumina HumanWG-6 v2 Expression BeadChips (Illumina, USA) and Illumina HumanHT-12 v4.0 Expression BeadChips (Illumina, USA) microarray platforms. For this, total RNA from snap frozen tissues was isolated using Trizol reagent (Thermo Fisher Scientific, USA), and further purified with RNeasy columns (Qiagen, Netherlands), and treated with DNase (RNase-free DNase Set, Qiagen, Netherlands; or DNase I, Invitrogen, Thermo Fisher Scientific, USA) to remove genomic DNA. The RNA concentrations were determined using Nanodrop ND-1000 spectrophotometer (Thermo Fisher Scientific, USA), and the quality of the RNA used was controlled using Experion^TM^ Automated Electrophoresis system (Bio-Rad Laboratories, USA), and the mean RQI value of all the samples were 7.5.

Microarray analysis was performed on samples obtained from 190 endometriotic lesions^26^ (76 peritoneal, 86 deep and 28 ovarian endometriosis) and from 101 endometrium biopsies of endometriosis patients and 43 endometrium biopsies of control women (Table 2). The hybridized images were scanned using Agilent’s microarray scanner and quantified with Feature Extraction Software (Agilent Technology, CA, USA). Raw intensity data was then globally normalized according to manufacturer’s instructions. Data from the Sentrix® Illumina HumanWG-6 v2 and Illumina HumanHT-12 v4.0 Expression BeadChips were loaded using *beadarray* R package^27^. For global correction, each chip generation was treated as a separate batch. Log transformation and quantile normalization was performed batch-wise using standard R Bioconductor methods^28–30^. We used the BLAST Method to map probes to their corresponding genes using up-to-date gene-to-probe associations all probe sequences were aligned to NCBI’s Nucleotide Sequence (*nt*) database^31^ adopting a procedure published in a previous study^32^. Since aligning to the *nt* database resulted in multiple hits across multiple species data was cleaned and filtered before being used to join the different array generations. To extract relevant features from the BLAST results data is annotated with up-to-date gene symbols and Entrez IDs. To achieve a more reliable annotation three different sources are used, dbOrg (https://biodbnet-abcc.ncifcrf.gov/db/dbOrg.php), HGNC (ftp://ftp.ebi.ac.uk/pub/databases/genenames/new/tsv/) and BioMart^33,34^. During the joining process, the symbol found in most of the annotation sources is used.

Combining the microarray data from the two Expression BeadChips data frames obtained from the BLAST approach are joined on the Entrez Gene ID and the RefSeq mRNA Accession ID, resulting in 27541 common probes corresponding to 24423 genes. To correct the variation originating in the different Expression BeadChips array versions the ComBat batch adjustment algorithm^35^ within the SVA R-Package^36^ was used. The quality of the merged data was then assessed by PCA and global correlation analysis.

## Data Records

The EndometDB is freely accessible at https://endometdb.utu.fi/. A copy of EndometDB is also made publicly available on figshare as a zip file containing a SQL dump of the database^26^ along with additional supplements data. All the raw data for the global transcriptomic data, generated by the Sentrix® Illumina HumanWG-6 v2 Expression BeadChips (Illumina, USA)^37^ and Illumina HumanHT-12 v4.0 Expression BeadChips (Illumina, USA)^38^ microarray platforms used in this study have been uploaded to the National Center for Biotechnology Information (NCBI) Gene Expression Omnibus (GEO) (https://www.ncbi.nlm.nih.gov/geo/). The normalized data from both the Sentrix® Illumina HumanWG-6 v2 Expression BeadChips (Illumina, USA) and the Illumina HumanHT-12 v4.0 Expression BeadChips (Illumina, USA) as well as the combined normalized data from both microarray platforms in the EndometDB have also been uploaded to GEO with the series accession number GSE141549^39^. The deposited data contains non normalized data matrix from both platforms as well as processed transcriptomic data files, together with the clinical features described in this report. This manuscript describes the samples, data collection, processing steps, and the EndometDB with freely available GUI for data analysis and interactive visualization.

## Technical Validation

### Quality control of RNA integrity

To determine RNA quality, Experion^TM^ Automated Electrophoresis system (Bio-Rad Laboratories, USA) was used. The integrity of RNA was calculated using RQI (RNA quality indicator) algorithm, where a high number indicates higher quality, with the maximum value being 10. The mean RQI value of all samples was 7.5 and the lowest acceptable RQI was > 6.

### Quality control of microarray profiling

The normalized RNA data was quality controlled using the ArrayQualityMetrics R package^40^.

### Validation of the microarray data using quantitative reverse transcription PCR (RT-qPCR)

**Fig. 6:**
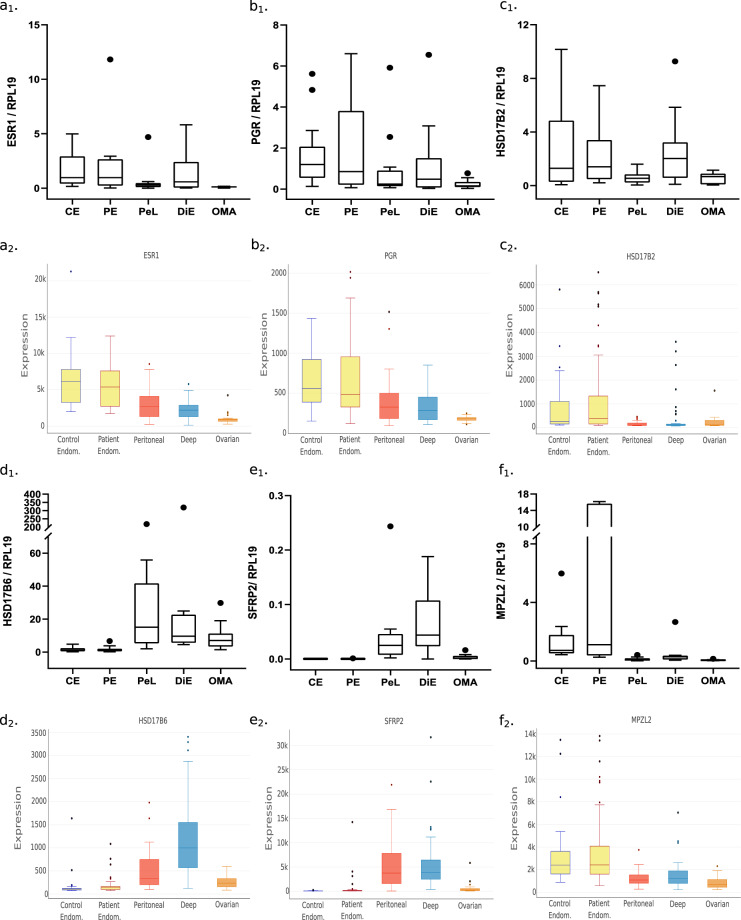
Validation of microarray result with qRT-PCR. Examples of the mRNA expression for steroid receptors (ESR1, PGR), steroid metabolizing enzymes (HSD17B2, HSD17B6), and WNT- pathway genes (SFRP2, and MPZL2) analyzed by RT-qPCR (a_1_, b_1_, c_1_, d_1_, e_1_, f_1_) and with the EndometDB (a_2_, b_2_, c_2_, d_2_, e_2_, f_2_).

To validate the transcriptomic data provided in the EndometDB by an independent method, we performed RT-qPCR analyses for various transcripts of selected enzymes involved in steroid synthesis, of certain androgen regulated genes and certain WNT signaling pathway genes^41–43^ (Online-only Table 1) in the ovarian, deep, peritoneal lesions and endometrium (Fig. 6). For those analyses we used 0,5 μg. of total RNA that was converted to cDNA using the DyNAmo HS SYBR Green 2-Step RT-qPCR kit (Finnzymes, Thermo Fisher Scientific, USA), followed by the qPCR reactions for 40 cycles with the primers presented in Table 4. Ribosomal protein L19 (RPL19) was used as reference gene for the data normalization. The RT-qPCR analyses were carried out in samples^26^ obtained from the proliferative and secretory phase samples of OMA (n = 10–18), DiE (n = 10–16), PeL (n = 10–19), and PE (n = 6–20). Endometrium and peritoneum of healthy women (CE, CP) and patients (PE, PP) were also included (n = 8–21). The expression ratio was calculated using the mathematical model for relative quantification in real-time PCR^44^. The ratio represents the factor by which the target gene of interest is expressed in endometriosis relative to patient eutopic endometrium after normalization to the reference gene.

**Table 4:** List of primers used for quantitative real-time PCR.

Primer name	Accession No.	Sense Primer (5′ → 3′)	Antisense Primer (5′ → 3′)	Target length
RPL19	NM_000981.4	AGGCACATGGGCATAGGTAA	CCATGAGAATCCGCTTGTTT	199
CYP19A1	NM_001347248.1	AGTGCATCGGTATGCATGAG	AGAAGGGTCAACACGTCCAC	205
HSD17B2	NM_002153.3	AACTGATGGGGAGCTTCTTCTTAT	CCTCCTCCCATGCTGCTGACA	147
HSD17B6	NM_003725.4	CTCCAGCATTCTGGGAAGAG	AATATGCTTGGGGGCTTCTT	217
ESR1	NM_000125.3	TGGATTTGACCCTCCATGAT	GATCTCCACCATGCCCTCTA	170
ESR2	NM_001437.2	TATCACATCTGTATGCGGAACC	TACATCCTTCACACGACCAGAC	225
AR	NM_000044.4	TGGCGGGCCAGGAAAGCGAC	GGGCAAAACATGGTCCCTGGCA	179
HGD	NM_000187.4	CTCTCAGGATCGGCTTTCAC	TGTCTCCAGCTCCACACAAG	244
MPZL2	NM_005797.4	GGGACAGATGCTCGGTTAAA	CAAGACACCCGGTCCTTAAA	173
PDGFRL	NM_006207.2	AAAAGTGGGGACGACATCAG	GGGAGATTCTCGTGGTGTGT	166
SMTN	NM_134270.2	GAGTCTGCCCAAGACCTCAG	AGTCTTGGCTCGACACCAGT	181
SRD5A3	NM_024592.5	TCCTTCTTTGCCCAAACATC	CTGATGCTCTCCCTTTACGC	211
TRH	NM_007117.5	CTGAAGCGTTGTGTGCAAAT	AGCCAGACACAGCACAACAC	204
STS	NM_000351.5	CATGGACATATTTCCTACAGTAGCC	GATCACGTCCATCAATGATCC	77
PRUNE2	NM_015225.3	CAGAAAACATGGAGCTGTGC	AAAGGGCTCCAGTTCTAGGC	80
DKK1	NM_012242.4	TCCGAGGAGAAATTGAGGAA	CCTGAGGCACAGTCTGATGA	157
DKK3	NM_015881.5	ACAGCCACAGCCTGGTGTA	CCTCCATGAAGCTGCCAAC	120
FZD7	NM_003507.1	GGCTGCGCTGCGAGAACTTC	CAGCGCGGTGAAGGGCAGGTC	146
FZD10	NM_007197.3	CCTCCAAGACTCTGCAGTCC	GACTGGGCAGGGATCTCATA	160
FRZB	NM_001463.4	GCAAGCAGTGAACGCTGTAA	GGCAGCCAGAGCTGGTATAG	214
HPRT1	NM_000194.3	TGCTCGAGATGTGATGAAGG	TCCCCTGTTGACTGGTCATT	192
SFRP1	NM_003012.5	CGAGTTTGCACTGAGGATGA	CAGCACAAGCTTCTTCAGGTC	130
SFRP2	NM_003013.3	CGAGGAAGCTCCAAAGGTAT	CTCCTTCACTTTTATTTTCAGTGCAA	112
WNT5A	NM_003392.4	TGGCTTTGGCCATATTTTTC	CCGATGTACTGCATGTGGTC	199
WISP2	NM_001323370.1	CTGTATCGGGAAGGGGAGAC	GGAAGAGACAAGGCCAGAAA	246

## Usage Notes

The EndometDB in its current form does not allow for others to add curated data of their own. However, we are open to adding data also from other groups in the field. To ensure that all investigators have an easy access to the data in our EndometDB, we developed a web application using HTML5, JavaScript, CSS, JS-libraries: jQuery, Plotly.js and R. Any internet enabled device using a modern browser can access the EndometDB (https://endometdb.utu.fi/). No user account needs to be created to access or use the features incorporated for exploration of the genes in the GUI. In exploring the EndometDB, users can:

1. View summary characteristics of the EndometDB.
2. Explore differentially expressed genes in endometrium, peritoneum, and endometriosis lesion.
3. Cluster genes across the above-mentioned tissues and lesions.
4. Explore how genes correlate with each other in the above-mentioned tissues and lesions.
5. Performing projections of data with PCA, MDS and LFDA.

## Data Availability

EndometDB uses open source components listed in the Table 3. Code for pre-processing of the data is available upon request. The Expression BeadChips were loaded using R function calls in the publicly available *beadarray* R package^27^. Log transformation and quantile normalization was performed using standard Bioconductor R packages^28–30^. The ComBat batch adjustment algorithm^35^ within the SVA R-Package^36^ was used to correct the variation in the different Expression BeadChips arrays. The EndometDB source code is available at our GitHub repository https://github.com/micawo/EndometDB.

## Online-only Table

**Online-only Table 1:** WNT pathway gene expression.

Gene ID	Main WNT pathway	Reference
SFRP2	Canonical	KEGG
GPC3	Canonical	1–3
FRZB	Canonical	KEGG
FZD7	Canonical	KEGG
RSPO3	Canonical	1–3
FZD4	Canonical	KEGG
DKK3	Canonical	KEGG
CAMK2G	Ca^2+^	KEGG
RSPO1	Canonical	1–3
PRKCB1	Ca^2+^	KEGG
JUN	Canonical	KEGG
NFATC1	Ca^2+^	KEGG
FOSL1	Canonical	KEGG
SFRP1	Canonical	KEGG
NKD2	Canonical	KEGG
PPP3CB	Ca^2+^	KEGG
MAPK10	PCP	KEGG
ROR1	PCP	1–3
SERPINF1	Canonical	KEGG
ROCK2	PCP	KEGG
WNT2B	Canonical	KEGG
PLCB2	Ca^2+^	KEGG
GPC6	Canonical	1–3
RSPO2	Canonical	1–3
CAMK2A	Ca^2+^	KEGG
NFATC4	Ca^2+^	KEGG
MYC	Canonical	KEGG
FZD1	Canonical	KEGG
GPC4	PCP	1–3
WNT5A	Canonical	KEGG
SOX17	Canonical	KEGG
NDP	Canonical	1–3
MMP7	Canonical	KEGG
WNT2	Canonical	KEGG
LGR4	Canonical	1–3
LEF1	Canonical	KEGG
FRAT2	Canonical	KEGG
CACYBP	Canonical	KEGG
CTNNBIP1	Canonical	KEGG
RUVBL1	Canonical	KEGG
SDC1	PCP	1–3
PTK7	Canonical	1–3
LGR5	Canonical	1–3
FZD3	Canonical	KEGG
FZD2	Canonical	KEGG
FZD10	Canonical	KEGG
RAC1	PCP	KEGG
WNT7A	Canonical	KEGG
GPC2	Canonical	1–3
TP53	Canonical	KEGG
WNT4	Canonical	KEGG
FZD9	Canonical	KEGG

PCP = planar cell polarity pathway, Ca^2+^pathway, 1 = {{41}} 2 = {{42}}, 3 = {{43}}
